# Screening for anxiety and depression among patients with cardiovascular diseases at a private tertiary care hospital in Karachi, Pakistan

**DOI:** 10.12669/pjms.41.6.7978

**Published:** 2025-06

**Authors:** Faryal Ghafor, Salma Rattani, Rozmin Jiwani, Zahra Tharani, Kashmira Nanji, Ahmed Sarki

**Affiliations:** 1 Faryal Ghafor, MScN , Aga Khan University School of Nursing and Midwifery, Karachi, Pakistan; 2 Salma Rattani, PhD Associate Professor, Aga Khan University School of Nursing and Midwifery, Karachi, Pakistan; 3 Rozmin Jiwani, PhD Associate Professor, UT Health San Antonio, School of Nursing, United States.; 4 Zahra Tharani, MScN Instructor, Kashmira Nanji, MS Epidemiology & Biostatistics Consultant / Freelance, Aga Khan University School of Nursing and Midwifery, Karachi, Pakistan; 5 Ahmed Sarki, PhD Assistant Professor, Aga Khan University School of Nursing and Midwifery, Uganda, Aga Khan University School of Nursing and Midwifery, Karachi, Pakistan

**Keywords:** Anxiety, Cardiovascular diseases, Depression, Frequency

## Abstract

**Background &Objective:**

Approximately 17.3 million individuals across the globe lose their lives to cardiovascular diseases with most of these cases occurring in low-to-middle-income countries. The incidence of cardiovascular disease in Pakistan is 918 cases per 100,000 individuals. Depression frequently impacts the health, expenses, and prognosis of patients with cardiovascular disease. This study aimed to screen anxiety and depression among patients with cardiovascular diseases.

**Method:**

A quantitative descriptive cross-sectional study was conducted at a tertiary care hospital in Karachi, Pakistan. Participants were patients with cardiac disorders and were receiving care in the inpatient unit or in the ambulatory clinic.

**Results:**

The study included 234 participants. Symptoms of anxiety and depression were screened in 28.6 percent participants and was significantly higher among single participants. Moreover, the presence of symptoms showed a significant difference based on gender, with a higher rate among females (p=0.025).

**Conclusion:**

The symptoms of anxiety and depression among patients with cardiovascular disease emphasizes the need for tailored interventions . These findings highlight the need to include regular screenings for anxiety and depression in cardiac care to better address the psychological challenges faced by vulnerable patient groups.

## INTRODUCTION

Cardiovascular diseases and depression have a bidirectional relationship.^1^Depression increases the risk of cardiovascular disease and worsens its outcomes.^2^ Depression screening should be performed in all adult patients with acute or chronic cardiovascular disease, this may also be helpful for younger individuals in the context of cardiovascular risk assessment.^3^,^4^ The American Heart Association (AHA) has advised screening for depression in cardiac patients, as depression and anxiety have been recognized as intimidating and negatively affecting prognosis in cardiac patients.^5^ People neglect the biomedical aspect of depression and consider it a normal response to stress-inducing scenarios.^2^ Cardiovascular diseases contribute 22.7% to the proportion of total deaths in Pakistan.^6^ One episode of depression enhances the risk of myocardial infarction fourfold.^7^ Depression in patients with cardiovascular disease (CVD) mostly remains undiagnosed. The health care providers show little concern about the psychological aspects of cardiac patients in Pakistan. The prevalence of mental health issues in South Asian countries, like India, Pakistan, and Bangladesh is less researched, and data and statistics results are taken from Western countries.^8^ This study aimed to screen anxiety and depression among patients with cardiovascular diseases and to explore the association between demographic factors and the symptoms of anxiety and depression.

**Fig.1:**
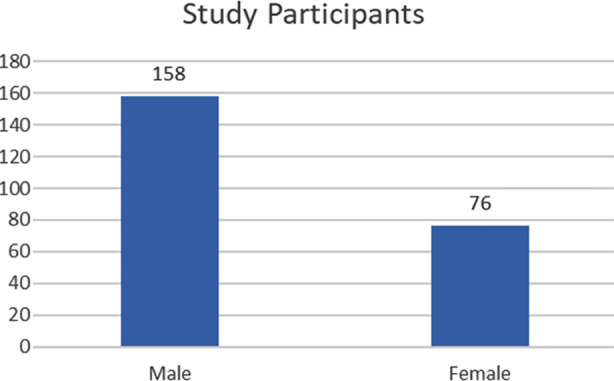
Study Participants (n=234). ***Note:*** This table presented gender distribution of study participants.

**Fig.2:**
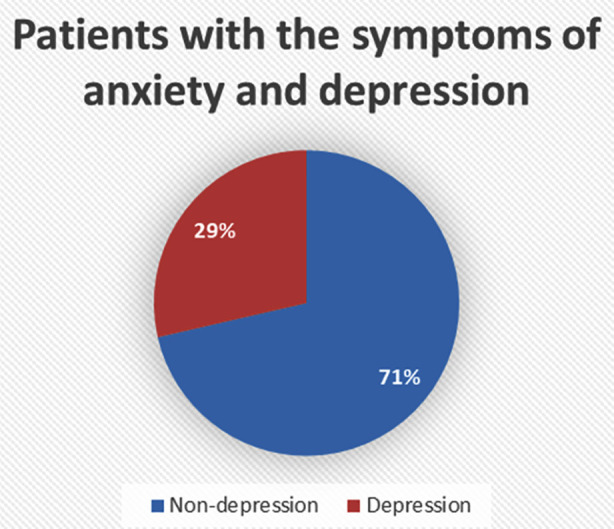
Frequency of patients with the symptoms of anxiety and depression

## METHODS

This quantitative descriptive cross-sectional study was conducted at a tertiary care hospital in Karachi, Pakistan. Data was collected between March to August 2023. Participants were patients with cardiac disorders and were receiving care in the inpatient unit or in the ambulatory clinic.

### Ethical statement:

The study obtained ethical approval (Study No. 2023-8092-24049, dated February 8, 2023) from the ethical review committee of the Aga Khan University a written informed consent was obtained individually from all participants before their involvement in the research.

### Sampling:

A non-probability purposive sampling method was used.

### Sample size calculation:

The software Open Epi Version three was used to calculate the sample size. With a 95% confidence level and 80% power, the sample size was determined to be 237 participants.

### Participants

Participants included 18 years and above male and female patients with cardiac disorders and were receiving care in the inpatient unit or in the ambulatory clinic. Patients with end-stage kidney diseases, liver cirrhosis, organ failure, cognitive impairment, history of drug or alcohol abuse, and those with unstable hemodynamic status were excluded from the study.

### Data collection:

Data was collected using two instruments: (1) Sociodemographic Questionnaire: This structured questionnaire captured participants’ demographic characteristics. (2) Aga Khan University Anxiety and Depression Scale (AKUDS): This scale, available in both English and Urdu, assessed depression with 25 items using Likert-scale responses reflecting experiences over the past two weeks. AKUDS has a sensitivity of 66%, a specificity of 79%, a positive predictive value of 83%, and a negative predictive value of 60%.

### Statistical analysis:

Descriptive statistics measured the frequency of patients with symptoms of anxiety and depression. The Mann-Whitney U test was used to compare the results between males and females. The Chi-square test assessed the significance of the association between participants’ demographic characteristics and anxiety and depression screening results. Univariate and multivariate logistic regression analyses were used to determine the strength of association between predictor variables and anxiety and depression.

## RESULTS

A total of 234 participants were enrolled in the study. Among these158 (67.5%) were males and (76) 32.5% were females. The majority of participants were between >60 to 70 years of age (34.2%). They were from different geographical areas, marital status and qualification. These details are presented in Table-III.

**Table-I:** Frequency of symptoms of Anxiety and Depression among Study Participants (n=234).

Variables	N	%	Median	Mean	Std. Deviation
AKUADS Score (0-75) Anxiety and Depression	___ 67	___ 28.6	14	15.82	9.579
Non-Anxiety and Depression	167	71.4	___	___	___

***Note:*** This table presented the mean scores for the symptoms of Anxiety and depression in study participants.

**Table-II:** Comparative Analysis of anxiety and depression scores across genders in AKUADS participants.

Gender		n	Mean Rank	Sum of Ranks	Mann Whitney	p-value
AKUADS	Male	158	112.14	17718.00	5157	0.026
	Female	76	128.64	9777.00		
	Total	234				

***Note:*** This table presented the difference in mean ranks of depression scores in males and females.

**Table-III:** Association between sociodemographic characteristics of participants and the presence of symptoms of Anxiety and depression (n=234).

Variable	Categories	f (%)	Depression	Non-Depression	P-value
Gender	Male	158(67.5)	38(56.7%)	120(71.9%)	.025
	Female	76(32.5)	29(43.3%)	47(28.1%)	
Age	Below 40 years	13(5.6)	6(9%)	7(4.20%)
	40 to 50 years	27(11.5)	9(13.40%)	18(10.80%)
	50 to 60 years	57(24.4)	11(16.40%)	46(27.50%)
	60 to 70 years	80(34.2)	17(25.40%)	63(37.70%)
	70 years and above	57(24.4)	24(35.80%)	33(19.80%)
Diagnosis	IHD	171(73.1)	53(79.1%)	118(70.7%)	.188
	Post-Surgery	63(26.9)	14(20.9%)	49(29.3%)	
Co-Morbids	Both DM, HTN	100(42.7)	32(47.8%)	68(40.7%)	.411
	DM	38(16.2)	7(10.4%)	31(18.6%)	
	HTN	66(28.2)	18(26.9%)	48(28.7%)	
	None	30(12.8)	10(14.9%)	20(12.0%)	
Residence	Punjab	6(2.6)	4(6%)	2(1.2%)	.146
	Sindh	197(84.2)	53(79.1%)	144(86.2%)	
	Balochistan	15(6.4)	6(9%)	9(5.4%)	
	KPK	8(3.4)	1(1.5%)	7(4.2%)	
	Others	8(3.4)	3(4.5%)	5(3%)	
Education	Primary	36(15.4)	19(28.4%)	17(10.2%)	.002
	Middle	15(6.4)	7(10.4%)	8(4.8%)	
	Matric	39(16.7)	11(16.4%)	28(16.8%)	
	Intermediate	33(14.1)	10(14.9%)	23(13.8%)	
	Bachelors	0.351	3.955	0.022	
	Masters	41(17.5)	8(11.9%)	33(19.8%)	
Marital Status	Married	213(91)	60(89.6%)	153(91.6%)	.801
	Single	21(9)	7(10.4%)	14(8.4%)	

The presence of symptoms of anxiety and depression among the study participants (n=234) was 28.6%. The median score of AKUADS was found to be 14, with a mean score of 15.82, with a standard deviation of 9.579 (Table-I). Female participants demonstrated significantly higher symptoms of anxiety and depression (mean rank = 128.64, p = 0.026) compared to male participants (Table-II).

There were significant associations of gender, age, and education level with the presence of anxiety and depression in patients with cardiovascular diseases. In all, 56.7% of males and 43.3% of females (p=0.025) experienced symptoms of anxiety and depression. Most of the participants who experienced symptoms were from the age group of 70 years and above (35.8%, p= 0.018). Moreover, 28.4% of the participants with primary education had experienced more symptoms as compared to those higher education levels (p=0.002). Other socio-demographic characteristics had insignificant relation (p=< 0.05) with the presence of symptoms (Table-III).

An intricate relationship was found between sociodemographic factors and the presence of symptoms of anxiety and depression. Firstly, concerning gender, females faced a statistically significant (OR = 1.948, p = 0.027) risk of developing symptoms of anxiety and depression. Single participants had an odds ratio of 1.275, as compared to married participants, but the results were insignificant (p=0.618). Participants below 40 years of age were found to be at higher risk of developing symptoms of anxiety and depression (OR:1.791, p:0.022) (Table-IV). However, multivariate logistic regression revealed that study participants with primary education (OR=4.283, p=0.026), and middle education (OR 3.29, p= 0.026) were more inclined to develop symptoms of anxiety and depression and the risk of developing symptoms of anxiety and depression decreased with an increase in education level (Table-V).

**Table-IV:** Association Between Sociodemographic Factors and Anxiety and Depression Risk.

Variable	Categories	f (%)	OR	Lower CI	Upper CI	p-value
Gender	Male ®	158(67.5)	0.317			0.027
	Female	76(32.5)	1.948	1.081	3.513	
Education	Primary	36(15.4)	4.610	1.675	12.687	0.004
	Middle	15(6.4)	3.609	1.009	12.917	
	Matric	39(16.7)	1.621	0.572	4.588	
	Intermediate	33(14.1)	1.793	0.614	5.236	
	Graduation	70(29.9)	0.853	0.317	2.300	
	Master®	41(17.5)	0.242			
Marital Status	Married ®	213(91)	0.392			0.618
	Single	21(9)	1.275	0.491	3.314	
	Below 40years	13(5.6)	1.179	0.351	3.955	0.022
Age	40 to 50years	27(11.5)	0.687	0.264	1.791	
	>50 to 60years	57(24.4)	0.329	0.142	0.763	
	>60 to 70years	80(34.2)	0.371	0.175	0.768	
	>70years and above ®	57(24.4)	0.727			
Diagnosis	IHD	171(73.1)	1.572	0.799	3.093	0.190
	Post-Surgery ®	63(26.9)	0.286			
Co-Morbids	Both DM and HTN	100(42.7)	0.941	0.395	2.241	0.422
	DM	38(16.2)	0.452	0.148	1.381	
	HTN	66(28.2)	0.750	0.295	1.906	
	None®	30(12.8)	0.500			
Residence	Punjab	6(2.6)	3.333	0.362	30.701	0.208
	Sindh	197(84.2)	0.613	0.142	2.656	
	Balochistan	15(6.4)	1.111	0.190	6.492	
	KPK	8(3.4)	0.238	0.019	3.011	
	Others	8(3.4)	0.600			

***Note:*** This table presented the multivariate regression analysis between socio-demographics and CVD in study participants.

**Table-V:** Multi-variate Logistic Regression Model.

Variable	Category	f (%)	OR	Lower CI	Higher CI	p-value
Education	Primary	36(15.4)	4.283	1.434	12.798	0.026
	Middle	15(6.4)	3.291	0.850	12.740	
	Matric	39(16.7)	1.625	0.547	4.832	
	Intermediate	33(14.1)	1.948	0.620	6.116	
	Graduation	70(29.9)	0.919	0.330	2.559	
	Master	41(17.5)				
Age	Below 40years	13(5.6)	1.077	0.290	3.994	0.043
	40 to 50years	27(11.5)	0.747	0.271	2.060	
	>50 to 60years	57(24.4)	0.334	0.138	0.807	
	>60 to 70years	80(34.2)	0.376	0.169	0.835	
	>70years and above	57(24.4)				
Gender	Male	158(67.5)				0.433
	Female	76(32.5)	1.310	0.667	2.570	

## DISCUSSION

The current study aimed to screen the patients with cardiovascular diseases for the symptoms of anxiety and depression. The findings indicate a significant association between cardiovascular diseases and anxiety and depression. This study found that symptoms of anxiety and depression is highly prevalent among patients with cardiovascular diseases , aligning with previous research.^9^–^13^ Depression prevalence among heart failure patients ranges from 20% to 40%, and in some cases, can be as high as 51.5% in low- and middle-income countries (LMICs).^14^,^15^ In these countries due to limited access to resources, socioeconomic status, and systemic discrimination anxiety and depression are more pronounced.^16^

This study screened the patients with cardiovascular diseases for the symptoms of anxiety and depression. These results were corresponding to the earlier studies. For instance, a study from the Faisalabad Institute of Cardiology in Pakistan reported a 79.5% prevalence of depression among patients with cardiovascular diseases, and a study in Trinidad and Tobago found a 40% prevalence among hospitalized cardiac patients.^10^,^17^

The global burden of depression, particularly among women and older adults with cardiovascular diseases, is exacerbated by factors such as financial stress, lack of social support, and chronic physical ailments.^18^,^19^ This highlights the critical need for integrated care approaches that address both physical and mental health in patients with cardiovascular diseases. Depression is a major global health issue, affecting 14% of the population and ranking fourth in its contribution to the overall disease burden.^20^ The prevalence is particularly high in LMICs, which bear over 80% of the global mental health burden.^21^ Significant regional disparities exist, with higher depression rates among myocardial infarction patients in Asia (45.03%) compared to North America (25.97%) and Europe/UK (23.50%). These differences are driven by socioeconomic, demographic, and cultural factors, underscoring the need for targeted interventions that consider the unique challenges of each region.^22^

Based on the screening tool the symptoms of anxiety and depressions were higher among the females but initial correlation between female gender and depression became insignificant upon controlling for education and age. This suggests that limited education and younger age, rather than gender itself, may contribute to depression in women. Other studies revealed gender and marital status significantly influence the prevalence and severity of depression among patients with cardiovascular disease.^22^–^24^ It is reported that women are more prone to depression due to lower levels of physical activity, which are linked to reduced self-efficacy and self-management capabilities, as well as experiencing cardiovascular diseases later in life, often with more comorbidities.^9^ However, in the current study no significant difference was found in symptoms of anxiety and depression between married and single individuals.

### Limitations:

This study lacks generalizability as it was only in one private hospital and participants were with specific sociodemographic characteristics. Comorbid included two; diabetes and hypertension and other comorbidities such as hypothyroidism, vitamin D deficiencies gut microbiome, reproductive and sexual disorders were not included. These factors could influence the presence and severity of anxiety and depression in patients with cardiovascular disease. These factors should be considered when interpreting the results and highlight the need for further research.

## CONCLUSION

This study emphasized the need for screening anxiety and depression in individuals with cardiovascular diseases, revealing a symptom rate of 28.6%, with significant differences based on gender, age, marital status, and educational level. The multidimensional nature of anxiety and depression in cardiovascular patients involves a complex interplay of sociodemographic and clinical factors, consistent with prior research. The study emphasizes the need for regular screening and gender-sensitive therapeutic practices due to the significant impact of anxiety and depression on overall health. Further extensive and longitudinal inquiries are necessary for a thorough understanding of anxiety and depression in this demographic, addressing the noted limitations. The findings highlight the importance of holistic healthcare, considering both physical and psychological well-being, to enhance patient care.

### Recommendations

Healthcare practitioners should consider the patients with cardiovascular disease be screened routinely for anxiety and depression. It is recommended that customized therapies be provided based on the individuals’ demographic characteristics and needs. Patient education programs raising awareness about psychological impacts, risk of depression, and accessible support resources are essential. Collaboration is needed between cardiology and psychological consultation for integrated care, advocating for policies and funding to support mental health treatments in cardiovascular care settings.

### Authors Contributions:

**FG:** Conceptualization, data collection, writing - original draft, writing - review and editing, and responsible for overall integrity of the study.

**SR:** Supervision, conceptualization, methodology, and feedback on manuscript drafts.

**RJ:** Writing - review and editing, and provision of frequent feedback on manuscript drafts.

**KN:** Formal analysis, data analysis, and software usage (SPSS).

**ZT and AS:** Review and provision of feedback on manuscript drafts.

All authors have read and approved the final version to be published.
